# Transplantation of Human Urine-Derived Stem Cells Ameliorates Erectile Function and Cavernosal Endothelial Function by Promoting Autophagy of Corpus Cavernosal Endothelial Cells in Diabetic Erectile Dysfunction Rats

**DOI:** 10.1155/2019/2168709

**Published:** 2019-09-09

**Authors:** Chi Zhang, Daosheng Luo, Tingting Li, Qiyun Yang, Yun Xie, Haicheng Chen, Linyan Lv, Jiahui Yao, Cuncan Deng, Xiaoyan Liang, Rongpei Wu, Xiangzhou Sun, Yuanyuan Zhang, Chunhua Deng, Guihua Liu

**Affiliations:** ^1^Department of Andrology, The First Affiliated Hospital of Sun Yat-sen University, Guangzhou, China; ^2^Reproductive Medicine Research Center, The Sixth Affiliated Hospital of Sun Yat-sen University, Guangzhou 510000, China; ^3^Department of Urology, Dongguan People's Hospital, Dongguan 523000, China; ^4^Guangdong Provincial Key Laboratory of Orthopedics and Traumatology, Guangzhou 510000, China; ^5^Department of Urology, The First Affiliated Hospital of Sun Yat-sen University, Guangzhou 510000, China; ^6^Wake Forest Institute for Regenerative Medicine, Wake Forest University, Winston-Salem, NC 27101, USA

## Abstract

**Aims:**

Cavernosal endothelial dysfunction is one of the factors in developing diabetic erectile dysfunction (DED), but the mechanism of cavernosal endothelial dysfunction is unclear. The present study is aimed at determining the contribution of autophagy in cavernosal endothelial dysfunction of DED rats and explaining the therapeutic effect of urine-derived stem cells (USCs).

**Methods:**

After rat corpus cavernosal vascular endothelial cells (CCECs) were isolated and cultured *in vitro*, CCECs were treated with advanced glycation end products (AGEs) to mimic the diabetic situation. Autophagy flux, proliferation, and apoptosis of CCECs were determined by mRFP-GFP-LC3 adenovirus infection combined with fluorescence observation and western blot analysis. USCs were isolated from the urine of six healthy male donors, and coculture systems of USCs and CCECs were developed to assess the protective effect of USCs for CCECs *in vitro*. The contribution of autophagy to the cellular damage in CCECs was evaluated by the autophagic inhibitor, 3-methyladenine (3-MA). Then, DED rats were induced by streptozotocin (50 mg/kg) and screened by apomorphine test (100 *μ*g/kg). In DED rats, USCs or PBS as vehicle was administrated by intracavernous injection (*n* = 15 per group), and another 15 normal rats served as normal controls. Four weeks after injection, erectile function was evaluated by measuring the intracavernosal pressure (ICP) and mean arterial pressure (MAP). Cavernosal endothelial function and autophagic activity were examined by western blot, immunofluorescence, and transmission electron microscopy.

**Results:**

*In vitro*, AGE-treated CCECs displayed fewer LC3 puncta formation and expressed less LC3-II, Beclin1, and PCNA but expressed more p62 and cleaved-caspase3 than controls (*p* < 0.05). Coculture of USCs with CCECs demonstrated that USCs were able to protect CCECs from AGE-induced autophagic dysfunction and cellular damage, which could be abolished by 3-MA (*p* < 0.05). DED rats showed lower ratio of ICP/MAP, reduced expression of endothelial markers, and fewer autophagic vacuoles in the cavernosal endothelium when compared with normal rats (*p* < 0.05). Intracavernous injection of USCs improved erectile function and cavernosal endothelial function of DED rats (*p* < 0.05). Most importantly, our data showed that the repaired erectile function and cavernosal endothelial function were the result of restored autophagic activity of the cavernosal endothelium in DED rats (*p* < 0.05).

**Conclusions:**

Impaired autophagy is involved in the cavernosal endothelial dysfunction and erectile dysfunction of DED rats. Intracavernous injection of USCs upregulates autophagic activity in the cavernosal endothelium, contributing to ameliorating cavernosal endothelial dysfunction and finally improving the erectile dysfunction induced by diabetes.

## 1. Introduction

Erectile dysfunction (ED) is a common complication of diabetes, affecting 35% to 90% of male patients [1]. Diabetic ED (DED) has an earlier onset and is more severe, and its incidence increases with disease duration [1–3]. The core pathogenesis of DED is cavernosal smooth muscle relaxation disorder and corporal fibrosis, leading to corporal veno-occlusive dysfunction [4–7]. Cavernosal endothelial dysfunction is currently suggested as an initiating factor in developing DED, and it is in the upstream of smooth muscle relaxation disorder and corporal fibrosis [8–10]. Cavernosal endothelial dysfunction can be promoted by hyperglycemia-induced formation of advanced glycation end products (AGEs) or increased oxidative stress [9, 11]. But the mechanism of cavernosal endothelial dysfunction remains to be elucidated.

Autophagy is an evolutionarily conserved cellular catabolic process, in which cytoplasmic materials are encased in intracellular vesicles and then delivered to lysosome for degradation [12, 13]. In most cells, a basal level of autophagy is occurring constantly and is essential to maintain the cellular homeostasis by eliminating damaged organelles, protein aggregates, and invading pathogens [14]. Numerous studies have indicated that autophagy plays critical and complex roles in diabetes and its complications [15–17]. Autophagy defect induced by mTORC1 upregulation was found in podocytes of both animal models and humans with diabetic nephropathy [18]. And it is reported that histone HIST1H1C regulates autophagy in the development of diabetic retinopathy [19]. However, limited studies have addressed the relationship between autophagy and DED.

Owing to the complex pathogenesis, DED does not respond well to phosphodiesterase type 5 inhibitors which are currently the first-line treatment for ED [20–22]. Consequentially, it is urgent to develop new therapies targeting DED. Recently, stem cell therapy has become a novel choice for ED treatment [23]. Several types of stem cells, such as bone marrow-derived mesenchymal stem cells and adipose tissue-derived stem cells, have been proven to be effective for the treatment of DED [24–26]. Urine-derived stem cells (USCs) are a new subpopulation of stem cells isolated from human urine. Our previous study and unpublished data revealed that USCs originate from parietal epithelium cells of the renal capsule [27]. They can be easily isolated and expanded by noninvasive method *in vitro*, possess multipotential differentiation capacity, and share similar characteristics of mesenchymal stem cells (MSCs) [28–30]. We have previously demonstrated that both USCs and USCs genetically modified with bFGF could improve erectile function and repair cavernosal endothelial structure in DED rat models [31]. The exact mechanisms of USCs' repair effect on cavernosal endothelial dysfunction and whether USCs take effect via regulating autophagic activity are unclear.

Based on the above evidence, in this study, we have focused on endothelial dysfunction in DED, and thus on the autophagic changes of cavernosal endothelial cells in a diabetic state *in vitro* and *in vivo*, and whether USCs restored erectile function and cavernosal endothelial function via the regulation of autophagy.

## 2. Materials and Methods

### 2.1. Isolation, Culture, and Identification of USCs

A total of 18 sterile voided urine samples (300-400 ml) from 6 healthy male volunteers (24-28 years old) were collected. The protocol to use human urine samples conforms to the ethical guidelines of Helsinki Declaration and was approved by the Institutional Review Board of the First Affiliated Hospital of Sun Yat-sen University. Written informed consent was obtained from every urine donor. The USCs were isolated and cultured as reported previously [28]. Briefly, fresh mid- and last-stream urine samples were centrifuged at 500 × g at room temperature for 10 minutes, and the cell pellet was gently suspended with mixed medium composed of keratinocyte serum-free medium and progenitor cell medium in a 1 : 1 ratio. The cell suspension was plated in 24-well plates and incubated at 37°C in a humidified atmosphere with 5% CO_2_. The culture medium was refreshed every other day. Colonies that derived from single cells were marked and passaged using 0.25% trypsin when they reached approximately 80% confluence. USCs at passage 3–4 were used for the following study.

The USCs were identified according to our previously described methods [31]. The osteogenic and adipogenic differentiation of the USCs were assessed by Alizarin red S (Santa Cruz Biotechnology, Santa Cruz, CA, USA) or Oil red O staining (Abcam, Cambridge, UK), respectively. Cell surface markers of USCs were measured with flow cytometry analysis (S3e™ Cell Sorter, Bio-Rad Laboratories, USA) by using fluorochrome-conjugated antibodies including CD24-FITC (20 *μ*l/test; 560992, BD Biosciences, USA), CD31-FITC (20 *μ*l/test; 560984, BD Biosciences), CD34-FITC (20 *μ*l/test; 560942, BD Biosciences), CD44-PE (20 *μ*l/test; 561858, BD Biosciences), CD45-PE (20 *μ*l/test; 561866, BD Biosciences), CD73-PE (20 *μ*l/test; 561014, BD Biosciences), CD90-APC (5 *μ*l/test; 561971, BD Biosciences), and CD105-PE (5 *μ*l/test; 560839, BD Biosciences).

### 2.2. Culture and Characterization of CCECs

The primary cultured rat corpus cavernosal vascular endothelial cells (CCECs) were purchased from Procell Life Science & Technology Co. Ltd. (Wuhan, China). CCECs were cultured in endothelial cell growth media (EGM-2; Lonza, Switzerland) at 37°C in a humidified atmosphere containing 5% CO_2_. The cells were passaged using 0.25% trypsin when they reached approximately 80% confluence. Passage 2 was used for the present study. The CCECs were identified by immunofluorescent staining of cell surface-bound CD31, as described previously [32].

### 2.3. Treatment of CCECs

CCECs at passage 2 were randomly divided into five groups: (1) control (homoculture of CCECs only); (2) bovine serum albumin (BSA; Abcam, USA); (3) AGE-BSA (AGEs; Abcam); (4) AGEs+USCs; and (5) AGEs+USCs+3-methyladenine (3-MA; Selleckchem, USA).

The coculture was performed in a Transwell unit (Costar 3413, Corning, USA). Cell suspensions of CCECs or USCs were prepared in endothelial cell growth media, respectively, at the concentration of 1 × 10^5^ cells/ml. CCECs (600 *μ*l) were cultured in the lower chamber, and USCs (100 *μ*l) were cultured in the upper chamber separately. After being incubated overnight, CCECs were infected with mRFP-GFP-LC3 adenovirus (HanBio Technology, Shanghai, China) according to the instructions. After 2 h, CCECs were changed with complete medium, and BSA/AGEs was added into the culture medium according to the grouping for 72 h. For groups (4) and (5), the upper chambers with USCs were placed above the lower CCEC chambers. For group (5), 3-MA was added into the lower chambers for 24 h. All experiments were performed in triplicate. The concentration of AGEs (200 *μ*g/ml) and 3-MA (2 mM) used in the present study was referred to previous studies [33–35].

### 2.4. Western Blot Analysis for Autophagy, Proliferation, and Apoptosis of CCECs after Treatment *In Vitro*

The autophagy, proliferation, and apoptosis of CCECs after treatment were analyzed by western blot as described previously [36]. Briefly, all the CCECs were harvested from each lower chamber, and proteins were extracted using RIPA lysis buffer (Cwbiotech, China) containing proteinase inhibitor cocktail (Cwbiotech) and phosphatase inhibitor cocktail (Cwbiotech). The primary antibodies included anti-PCNA (1 : 1000; 200947-2E1, ZenBio, China), anti-cleaved-caspase3 (1 : 1000; 9661, Cell Signaling Technology, USA), anti-LC3 A/B (1 : 1000; 4108, Cell Signaling Technology), anti-p62 (1 : 1000; ab56416, Abcam), anti-Beclin1 (1 : 2000; ab207612, Abcam), and anti-GAPDH (1 : 2000; Affinity Biosciences, USA) antibodies. PCNA is an indicator of cell proliferation and cleaved-caspase3 is an indicator of cell apoptosis. LC3, p62, and Beclin1 are autophagic markers. GAPDH was used as loading control.

### 2.5. Autophagic Flux Assay of CCECs after Treatment *In Vitro* via Confocal Microscopy

The fluorescent signal of GFP could be quenched under the acidic condition, but the mRFP fluorescent signal has no significant change. The neutral autophagosomes are shown as yellow puncta (RFP+GFP+), and acidic autolysosomes are shown as red puncta (RFP+GFP-) [37]. The mRFP-GFP-LC3 adenovirus makes it possible to monitor the progression of autophagic flux.

The LC3 puncta were examined with a confocal microscope (TSC-SP8, Leica, Germany). For each group, ten independent images were randomly selected to count the number of LC3 puncta.

### 2.6. Establishment of a DED Rat Model and USC Implantation *In Vivo*

Sixty-five male Sprague-Dawley rats (8–10 weeks old) were used in this study. The animal procedures were approved by the Institutional Animal Care and Use Committee of Sun Yat-sen University. Diabetes was induced by intraperitoneal (ip) injection of streptozotocin (STZ; 50 mg/kg; Sigma-Aldrich, USA) dissolved in pH 4.5 citric acid buffer. Diabetes was defined as a random blood glucose level higher than 300 mg/dl for three consecutive days after 72 hours of STZ injection. Eight weeks after STZ injection, an apomorphine (Sigma-Aldrich) test (100 *μ*g/kg) was performed to confirm DED rats according to Heaton's method [38].

After being anesthetized with pentobarbital sodium (50 mg/kg, ip), the DED rats received bilateral intracavernous injection of a total of 1 × 10^6^ USCs in 200 *μ*l phosphate-buffered saline (USC-treated group) or just 200 *μ*l PBS (DED group) (*n* = 15 per group), as our previous study described [24]. Another 15 normal rats served as the control group.

### 2.7. Erectile Function Evaluation

Erectile function was evaluated via intracavernosal pressure (ICP) and the ratio of ICP to mean arterial pressure (MAP) at four weeks after intracavernous injection, as previously described [39]. Briefly, rats were anesthetized with pentobarbital sodium (50 mg/kg, ip). The left carotid artery was cannulated with a PE-50 catheter filled with heparinized saline (250 IU/ml) to monitor the MAP. A 25-gauge needle filled with heparin (250 IU/ml) was inserted into the penile crus and connected to another pressure transducer. The cavernosal nerve was isolated and hooked by a bipolar electrode 3–4 mm distal to the major pelvic ganglion. Monophasic rectangular pulses (stimulus parameter settings of 2 ms width, 5 V voltage, 25 Hz frequency, and 60 s duration) were delivered from the stimulator (BL-420F, Taimeng, China). Three electrostimulations were replicated at intervals of 10 minutes. MAP and ICP were recorded and analyzed with BL New Century 2.1 software (Taimeng). The erectile function was evaluated as the ratio of ICP/MAP to normalize for variations in systemic blood pressure. The penis was then harvested for histological and western blot analysis.

### 2.8. Western Blot Analysis for Endothelial Function and Autophagy in Corpus Cavernosum Tissues

The corpus cavernosum tissues were lysed, and western blot analysis was performed as described previously [36]. The primary antibodies included anti-CD31 (1 : 2000; ab222783, Abcam), anti-eNOS (1 : 500; ab76198, Abcam), anti-phosphor-eNOS (S1177) (1 : 1000; 9571, Cell Signaling Technology), anti-VEGFRA (1 : 200; ab1316, Abcam), anti-VEGFR2 (1 : 1000; 9698, Cell Signaling Technology), anti-LC3 A/B (1 : 1000; 4108, Cell Signaling Technology), anti-p62 (1 : 1000; ab56416, Abcam), anti-Beclin1 (1 : 2000; ab207612, Abcam), and anti-GAPDH (1 : 2000; Affinity Biosciences, USA) antibodies. CD31, eNOS, phosphor-eNOS (S1177), VEGFRA, and VEGFR2 are indicators of endothelial function.

### 2.9. Immunofluorescent Staining Analysis for Endothelial Marker in Corpus Cavernosum Tissues

The corpus cavernosum tissues were fixed in 4% paraformaldehyde overnight. Paraffin-embedded tissue specimens were routinely prepared, sectioned at 5 *μ*m thickness. To visualize the tissular expression of endothelial marker, an anti-CD31 antibody (1 : 100; ab222783, Abcam) was used for immunofluorescent staining, as described previously [36]. Images were captured with a confocal microscope (TSC-SP8, Leica).

### 2.10. Autophagic Vacuole Observations via Transmission Electron Microscopy

Specimens of the corpus cavernosum were fixed in a fixative for TEM (Servicebio, China) at 4°C for 2-4 h and then postfixed in osmium tetroxide and embedded in EMBed 812 (SPI, USA). The specimens were cut into 0.1 *μ*m sections, stained with uranyl acetate/lead citrate, and viewed with a transmission electron microscope (TEM; HT7700, Hitachi, Japan). The tissues obtained from three rats in each group were examined. For each specimen, ten cavernosal endothelial cells were randomly selected, and the average number of autophagic vacuoles (including autophagosomes and autolysosomes) was compared between the samples collected from each group.

### 2.11. Statistical Analyses

Continuous values were expressed as mean ± standard deviation. One-way analysis of variance followed by a Student-Newman-Keuls post hoc test for multiple comparisons was used when appropriate. A two-tailed *p* < 0.05 was considered as statistically significant. Statistical analysis was performed with IBM SPSS Statistics 23.0 (IBM, USA).

## 3. Results

### 3.1. Characterization of USCs

**Figure 1:**
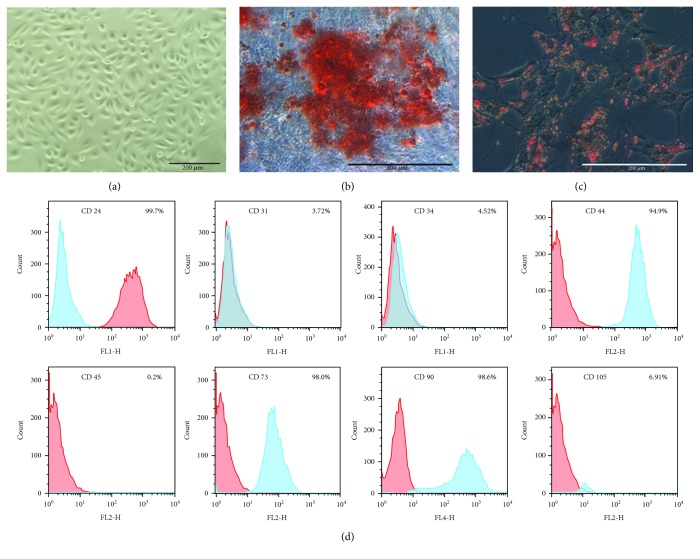
Characterization of urine-derived stem cells (USCs). (a) The typical rice-shaped appearance of USCs (p1). (b) Osteogenic- and (c) adipogenic-induced USCs with Alizarin red S staining or Oil red O staining, respectively. (d) Flow cytometry analysis showed that USCs (p3) were strongly positive for mesenchymal stem cell markers (CD24, CD44, CD73, and CD90), weakly positive for CD105, and negative for hematopoietic stem cell markers (CD31, CD34, and CD45).

USCs exhibited typical rice-shaped appearance (Figure 1(a)). The osteogenic- and adipogenic-induced USCs (stained with Alizarin red S or Oil red O, respectively) confirmed the multipotential differentiation capacity of USCs (Figures 1(b) and 1(c)). Flow cytometry analysis showed that USCs were strongly positive for MSCs markers (CD24, CD44, CD73, and CD90), weakly positive for CD105, and negative for hematopoietic stem cell markers (CD31, CD34, and CD45) (Figure 1(d)).

### 3.2. Characterization of CCECs

**Figure 2:**
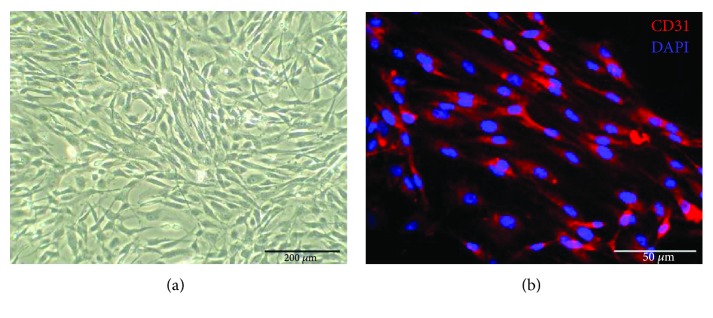
Characterization of corpus cavernosal vascular endothelial cells (CCECs). (a) The typical cobblestone-like appearance of CCECs (p1). (b) Immunofluorescent staining showed that more than 90% of CCECs (p2) were positive for endothelial markers (CD31, red fluorescence).

CCECs exhibited typical cobblestone-like appearance (Figure 2(a)), whose morphology and growth features were similar to previous reports [40, 41]. Immunofluorescent staining was performed to analyze the expression of endothelial marker (CD31), and more than 90% of CCECs stained positive for CD31 (Figure 2(b)).

### 3.3. AGEs Induce Autophagic Dysfunction and Cellular Damage in CCECs *In Vitro*

**Figure 3:**
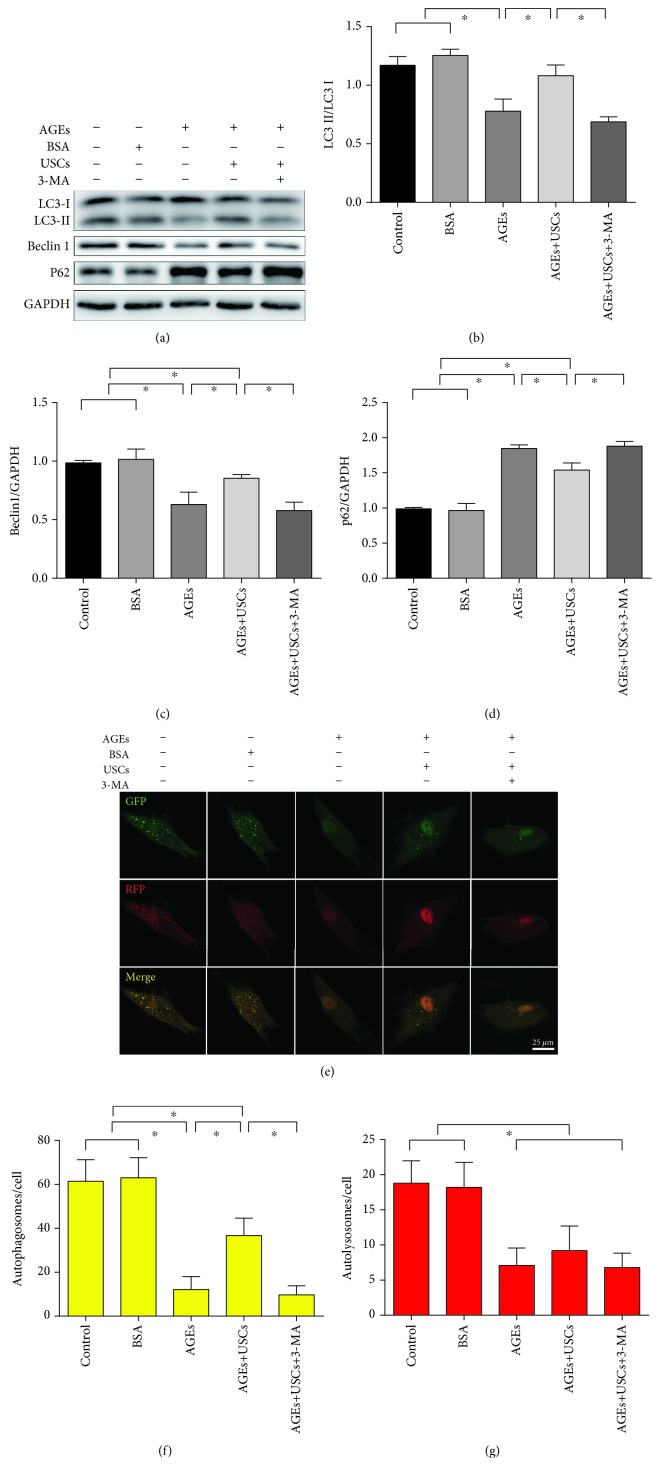
USCs protected CCECs from AGE-induced autophagic dysfunction in vitro and inhibition of autophagy attenuated the protective effect of USCs. (a) Western blot and (b–d) quantification for western blot of autophagy positive-related proteins (LC3 and Beclin1) and autophagy negative-related proteins (p62) in CCECs for each group. (e) Representative images of LC3 staining in CCECs for each group after infection with mRFP-GFP-LC3 adenovirus. Autophagosomes were shown as yellow puncta (RFP+GFP+), and autolysosomes were shown as red puncta (RFP+GFP-). (f, g) Quantification for autophagosome and autolysosome formation representing puncta staining sites per cell of 30 cells from each group. Treatment groups: control, BSA, AGEs, AGEs+USCs, and AGEs+USCs+3-MA. The concentration of BSA or AGEs was 200 *μ*g/ml. The concentration of 3-MA was 2 mM, and the treat time was 24 h. *n* = 3. ^∗^*p* < 0.05. C-caspase3: cleaved-caspase3; BSA: bovine serum albumin; AGEs: advanced glycation end products; 3-MA: 3-methyladenine.

Western blot analysis was performed to assess autophagic markers in protein levels. Compared with blank controls, AGE treatment significantly reduced the ratio of LC3-II/LC3-I and the expression of Beclin1 in CCECs and simultaneously increased the level of autophagic substrate (p62) (*p* < 0.05) (Figures 3(a)–3(d)). To further confirm the effect of AGEs on autophagic flux, we examined the mRFP-GFP-LC3 puncta formation with a confocal fluorescence microscope. Consistently, CCECs treated with AGEs displayed significantly reduced number of both autophagosomes (yellow puncta) and autolysosomes (red puncta) (*p* < 0.05) (Figures 3(e)–3(g)).

**Figure 4:**
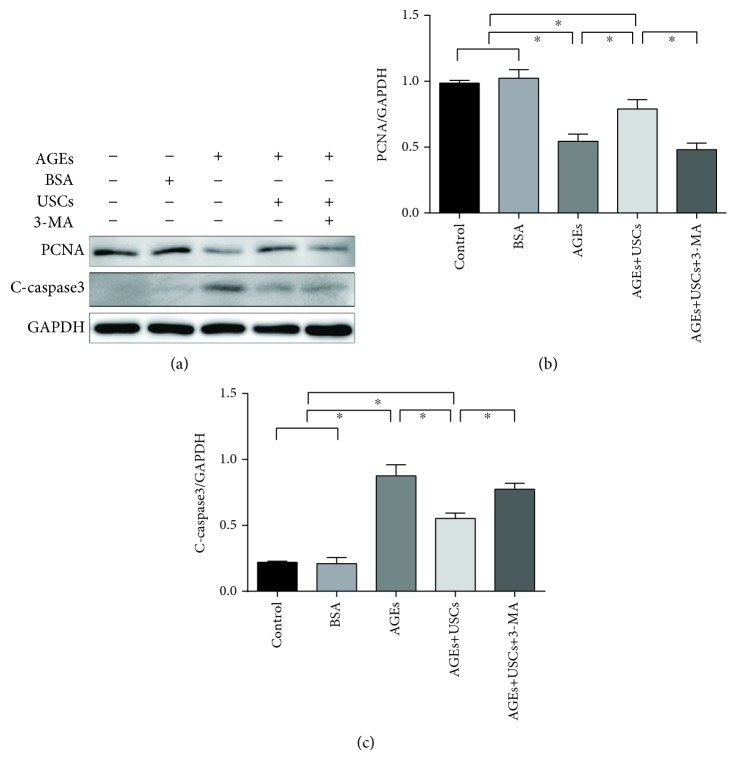
USCs protected CCECs from AGE-induced cellular damage in vitro and inhibition of autophagy attenuated the protective effect of USCs. (a) Western blot and (b, c) quantification for western blot of proliferation-related protein (PCNA) and apoptosis-related protein (C-caspase3) in CCECs for each group. Treatment groups: control, BSA, AGEs, AGEs+USCs, and AGEs+USCs+3-MA. The concentration of BSA or AGEs was 200 *μ*g/ml. The concentration of 3-MA was 2 mM, and the treat time was 24 h. *n* = 3. ^∗^*p* < 0.05. C-caspase3: cleaved-caspase3; BSA: bovine serum albumin; AGEs: advanced glycation end products; 3-MA: 3-methyladenine.

Cellular viability and apoptosis of CCECs were determined via western blot analysis. As shown in Figures 4(a)–4(c), AGEs significantly decreased the expression of proliferation-related protein PCNA and increased the expression of apoptosis-related protein cleaved-caspase3 (*p* < 0.05), which indicated that AGEs induced cellular damage in CCECs.

Compared with the control group, none of the above changes was observed following BSA treatment (*p* > 0.05), indicating that BSA was not cytotoxic to CCECs (Figures 3(a)–3(g) and 4(a)–4(c)).

### 3.4. USCs Protect CCECs from AGE-Induced Autophagic Dysfunction and Cellular Damage *In Vitro*

Compared with the AGE-treated group, the ratio of LC3-II/LC3-I was higher, the expression of Beclin1 was increased, and the protein levels of p62 were reduced in the USC coculture group demonstrated by western blot analysis (*p* < 0.05) (Figures 3(a)–3(d)). Moreover, autophagic flux detection via mRFP-GFP-LC3 adenovirus showed that USCs markedly increased the number of autophagosomes in AGE-treated CCECs (*p* < 0.05). Interestingly, the autolysosomes displayed an increasing tendency, but it was not statistically significant (*p* > 0.05) (Figures 3(e)–3(g)).

Importantly, when cocultured with USCs, the expression of PCNA was significantly reduced and the expression of cleaved-caspase3 was increased in AGE-treated CCECs (*p* < 0.05) (Figures 4(a)–4(c)). These data indicated that the USCs could protect CCECs from AGE-induced autophagic dysfunction and cellular damage.

In order to further confirm the protective effect of USCs, specific autophagy inhibitor (3-MA) was added in the medium of CCECs. Our data showed that the inhibition of autophagy by 3-MA could abolish the protective effect of USCs (Figures 3(a)–3(g) and 4(a)–4(c)).

### 3.5. Autophagic Activity Is Decreased in Cavernosal Endothelium of DED Rats

**Figure 5:**
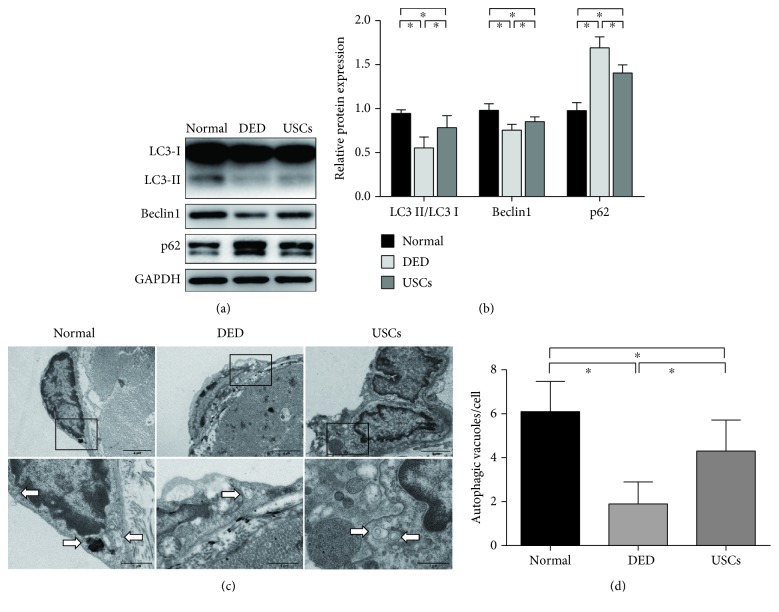
USCs restored autophagic activity of the cavernosal endothelium in DED rats. (a) Western blot and (b) quantification for western blot of autophagy positive-related proteins (LC3 and Beclin1) and autophagy negative-related proteins (p62) in cavernous tissue of normal rats and DED rats 4 weeks after intracavernous injection of PBS or USCs (*n* = 6 per group). (c) Representative TEM images of autophagic vacuoles (arrows) in cavernosal endothelial cells for each group. (d) Quantification for average autophagic vacuoles in 10 cells randomly selected from every specimen in each group (*n* = 3 per group). ^∗^*p* < 0.05. TEM: transmission electron microscopy.

Importantly, significantly lower ratio of LC3-II/LC3-I and expression of Beclin1 and higher levels of p62 were found in corpus cavernosum tissues of the DED group than in the normal control group by western blot analysis (*p* < 0.05) (Figures 5(a) and 5(b)). To further investigate the exact autophagic activity in the cavernosal endothelium, autophagic vacuoles were directly observed via TEM. The number of autophagic vacuoles in DED rats' cavernosal endothelial cells was significantly smaller than that in the normal control group (*p* < 0.05) (Figures 5(c) and 5(d)).

### 3.6. USCs Improve Erectile Function and Cavernosal Endothelial Function in DED Rats

**Figure 6:**
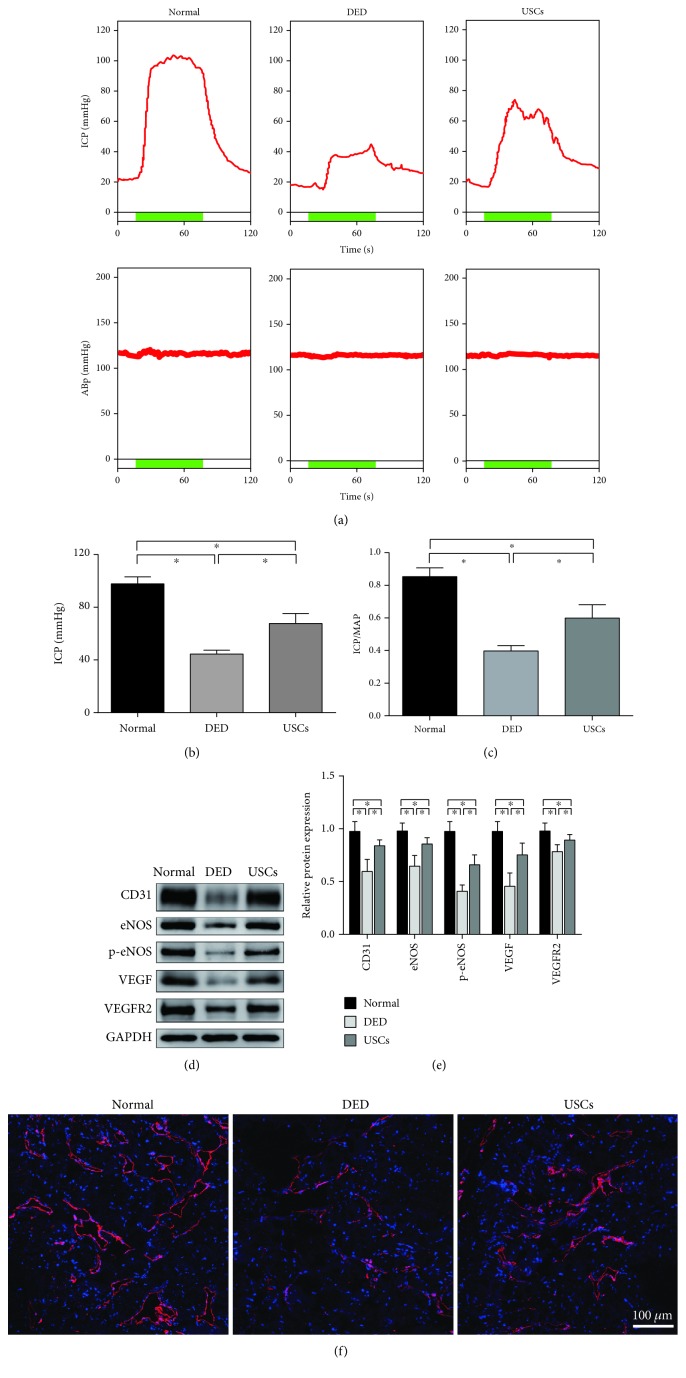
USCs improved erectile function and cavernosal endothelial function in diabetic erectile dysfunction (DED) rats. (a) Representative ICP tracing response to the stimulation of cavernous nerve (2 ms, 5 V, 25 Hz, and duration of 60 s) in normal rats and DED rats 4 weeks after intracavernous injection of PBS or USCs. (b) The USC injection increased ICP of DED rats, and (c) the ratio of ICP to MAP was calculated for each group (*n* = 8 per group). (d) Western blot and (e) quantification for western blot revealed that CD31, eNOS, p-eNOS, VEGFRA, and VEGFR2 expressions were all increased in the USC-treated group 4 weeks after cell transplantation (*n* = 6 per group). (f) Immunofluorescent staining confirmed the significantly higher expression of CD31 in cavernous tissue. ^∗^*p* < 0.05. ICP: intracavernous pressure; MAP: mean arterial pressure; p-eNOS: phospho-eNOS.

As shown in Figures 6(a)–6(c), four weeks after USC intracavernous injection, the ICP and ICP/MAP ratio of the USC-treated group reached up to 67.6 ± 7.6 mmHg and 60.1 ± 8.3%, respectively, which were significantly higher than those of the DED group (44.5 ± 3.0 mmHg and 39.8 ± 3.4%) (*p* < 0.05), representing an improved erectile function. But the values were still lower than those in the normal control group (97.7 ± 5.3 mmHg and 85.7 ± 5.4%) (*p* < 0.05).

A series of endothelial markers were assessed by western blot analysis (Figures 6(d) and 6(e)). The results showed a large decrease in CD31, eNOS, phospho-eNOS, VEGFRA, and VEGFR2 expression in DED rats compared with the normal control group (*p* < 0.05). Intracavernous injection of USCs partially restored the endothelial content in DED rats' penile tissues (*p* < 0.05). Similarly, the expression of CD31 decreased in DED rats' penile tissues and increased in USC treatment confirmed by immunofluorescent staining analysis (Figure 6(f)).

### 3.7. USCs Restore Autophagic Activity of Cavernosal Endothelium in DED Rats

Compared with the DED group, the USC-treated group exhibited significantly higher ratio of LC3-II/LC3-I, higher levels of Beclin1 in the cavernous tissue, and lower levels of p62 (*p* < 0.05) (Figures 5(a) and 5(b)). Correspondingly, more autophagic vacuoles were observed via TEM in DED rats' cavernosal endothelium after USC injection than in the DED group (*p* < 0.05) (Figures 5(c) and 5(d)). Taken together, these observations indicated that USCs could restore autophagic activity in the cavernosal endothelium of DED rats.

## 4. Discussion

The present study demonstrated that AGEs could inhibit the autophagic flux in CCECs and consequently lead to cellular damage *in vitro*. After being cocultured with USCs, the autophagic activity of CCECs was restored and cellular damage was alleviated. In further experiment, we found that suppressed autophagic activity was involved in the dysfunction of cavernosal endothelial cells in DED models. Intracavernous injection of USCs led to a significant increase of autophagic activity in the cavernosal endothelium, which coincided with improved cavernosal endothelial function and erectile function.

AGEs are a group of heterogeneous compounds continuously formed under hyperglycemic conditions [42]. Increased levels of AGEs have been found in both diabetic human beings and rodents [34, 43]. There is evidence that the accumulation of AGEs is associated with diabetic complications [11] and is responsible for cavernosal endothelial dysfunction in DED [9, 11]. AGEs have been adopted in some previous studies [32–34] to investigate the cellular effects of chronic hyperglycemia, as a more appropriate treatment factor in *in vitro* experiment than just high glucose level. Our data showed that, after treatment with AGEs, CCECs displayed reduced number of autophagosomes and autolysosomes, reduced expression of autophagy positive-related proteins (LC3 and Beclin1), and increased expression of autophagy negative-related protein (p62), indicating a blocked autophagic flux. At the same time, the cellular viability was decreased and apoptosis was increased, revealing the AGE-induced autophagic dysfunction was relevant to cellular damage. Similar outcomes were reported in the AGE-treated podocytes and chondrocytes from previous studies [33, 34].

However, in some other studies, AGEs induced cellular autophagy, which is contrary to our results [32, 44]. There are likely several explanations for this contradiction. On the one hand, different types of cells have different sensibility and different autophagic response to AGEs. On the other hand, the concentration of AGEs and exposure time determine the treatment effects. Short exposure to AGEs causes the cells to be in a state of acute stress, which is a common trigger of autophagy, activating the cellular self-protection program [12]. But a high concentration of AGEs and long exposure time tend to cause cellular decompensation, when autophagic dysfunction and cellular damage are observed. These concentration-dependent and time-dependent effects have already been demonstrated in previous study [33, 34].

Then, we established a DED rat model to further confirm the above phenomenon *in vivo*. Besides a lower ratio of ICP/MAP and decreased expression of a series of endothelial function-related proteins in DED rats, levels of autophagic protein markers (LC3, p62, and Beclin1) in corpus cavernosum tissues of DED rats indicate a reduced autophagic activity, which is in accordance with other studies [45]. But these results only represented the autophagic change in the whole corpus cavernosum, and the endothelium only accounts for a small proportion of penile tissue. To detect the autophagic activity exactly in the endothelium, we used TEM to observe the autophagic vacuoles in endothelial cells. We found that the number of autophagic vacuoles in DED rats' cavernosal endothelial cells was significantly fewer than that in the normal control group. Taking all the data of *in vitro* and *in vivo* experiments into consideration, we have sufficient reasons to speculate that long-standing diabetes induces autophagic dysfunction in cavernosal endothelial cells, eventually leading to endothelial dysfunction and erectile dysfunction in DED rat. In addition, it is likely that the autophagy in the cavernosal smooth muscle also plays a significant role in the condition of DED, and more studies are needed to examine its relative impact vis-à-vis the endothelium.

As we have revealed the autophagic disorder in the cavernosal endothelium of DED rats, the regulation of autophagy may be a new therapeutic target for reversing DED. It has been reported that rapamycin can ameliorate erectile function via inducing autophagy in DED rats [46]. Nevertheless, autophagy possesses organ specificity, tissue specificity, and even cell specificity, that is, a different body part has different autophagic changes in the same disease. Moreover, excessively activated autophagy would lead to negative impacts [47]. Therefore, systemic administration of autophagy-regulated agents is inappropriate. In recent years, stem cell therapy emerges as a promising strategy in treating chronic diseases [48, 49]. Stem cells can help repair damaged tissues or structures towards the normal state, and numerous studies have demonstrated that stem cells take effect via restoring autophagy homeostasis [50, 51]. Several types of MSCs, such as bone marrow-derived mesenchymal stem cells and adipose tissue-derived stem cells, have been utilized to treat DED via intracavernous injection and shown positive effects [24, 25]. However, these stem cells must be obtained by invasive methods, thus limiting their use in clinical practice. USCs may be a better cell candidate as they can be obtained through a safe, simple, low-cost, and noninvasive procedure.

In this study, we utilized a coculture system to investigate the effects of USCs on CCECs. We found that USCs could partly correct the autophagic disorder of AGE-treated CCECs and reduce cellular damage *in vitro*, and these protective effects could be abolished by autophagy inhibitors. Furthermore, we found that intracavernous injection restored autophagic activity in cavernosal endothelial cells of DED rats, as well as the cavernosal endothelial function and erectile function. These outcomes contribute to a better understanding of the mechanism of USCs in the treatment of DED. Our previous outcomes showed that rare labeled USCs could be tracked in the penile tissue since day 7 after cell transplantation, which was similar to most studies that utilized other MSCs to treat ED rat models [31]. Thus, we speculate that it was the paracrine effect of USCs that regulated autophagic balance due to USCs were able to secrete lots of paracrine factors [30], and further studies are needed to verify this hypothesis. It is reported that VEGF expressed by MSCs can take part in autophagy via triggering the PI3K/AKT/mTOR signaling pathway and consequently ameliorate DED in rats [45]. Maybe USCs regulate autophagy via the same signaling pathway, as we have demonstrated that VEGF is one of the growth factors that USCs secrete [31].

Due to the fact that human cells were injected into immunocompetent rats, the issue of immune tolerance is very important. As a type of MSCs, USCs possess the same immunomodulatory and immunosuppressive property, which permits their allogeneic or even xenogeneic transplantation into immunocompetent recipients in the absence of immunosuppressants [52]. Our previous studies have proven that neither immune reaction nor inflammatory response occurred within the injected sites of rats' penile tissue after injection of USCs [31, 36]. As for the limitation of USCs, their therapeutic effect may be influenced by changes in the biochemical composition of urine. Thus, for patients with urological diseases, such as urinary infection and renal or other urologic neoplasms, their USCs do not appear to be suitable for autologous cell transplantation.

Inevitably, some limitations existed in our study. First, the exact autophagic pathway that is abnormal in DED and by which USCs restore autophagic activity of the cavernosal endothelium needs to be clarified in further studies. Second, no attempt was done to assess changes in the functions of cavernosal smooth muscle, fibroblasts, or peripheral nerve after endothelial dysfunction was alleviated. Third, the effects of USCs are complicated, and it is unclear how long the USCs persist, when the paracrine effects occur, and how long the effects last.

## 5. Conclusion

Our study suggests that impaired autophagy is involved in the cavernosal endothelial dysfunction and erectile dysfunction of DED rats. Intracavernous injection of USCs upregulates autophagic activity in the cavernosal endothelium, contributing to ameliorating cavernosal endothelial dysfunction and finally improving the erectile dysfunction induced by diabetes. These findings provide a basis for the future use of USCs as a new biological therapeutic approach for DED.

## Data Availability

The data are available by contacting the corresponding authors.
